# Priorities for implementation research on diagnosing cancer in primary care: a consensus process

**DOI:** 10.1186/s12913-023-10330-z

**Published:** 2023-11-27

**Authors:** Thomas A Willis, Richard D Neal, Fiona M Walter, Robbie Foy

**Affiliations:** 1 Leeds Institute of Health Sciences, University of Leeds, Clarendon Way, Leeds, LS2 9NL United Kingdom; 2 Department of Health and Community Sciences, Faculty of Health and Life Sciences, University of Exeter, St Luke’s Campus Heavitree Road, Exeter, EX1 2LU UK; 3 Wolfson Institute of Population Health, Barts and the London School of Medicine and Dentistry, Queen Mary University of London, London, UK

**Keywords:** Cancer, Consensus, Early diagnosis, Expert panel, Primary care, Research priorities

## Abstract

**Background:**

The early detection and diagnosis of cancer to reduce avoidable mortality and morbidity is a challenging task in primary health care. There is a growing evidence base on how to enable earlier cancer diagnosis, but well-recognised gaps and delays exist around the translation of new research findings into routine clinical practice. Implementation research aims to accelerate the uptake of evidence by health care systems and professionals. We aimed to identify priorities for implementation research in early cancer diagnosis in primary care.

**Methods:**

We used a RAND/UCLA modified Delphi consensus process to identify and rank research priorities. We asked primary care physicians, patients and researchers to complete an online survey suggesting priorities for implementation research in cancer detection and diagnosis. We summarised and presented these suggestions to an 11-member consensus panel comprising nine primary care physicians and two patients. Panellists independently rated the importance of suggestions on a 1–9 scale (9 = very high priority; 1 = very low priority) before and after a structured group discussion. We ranked suggestions using median ratings.

**Results:**

We received a total of 115 suggested priorities for implementation research from 32 survey respondents (including 16 primary care professionals, 11 researchers, and 4 patient and public representatives; 88% of respondents were UK-based). After removing duplicates and ineligible suggestions, we presented 37 suggestions grouped within 17 categories to the consensus panel. Following two rounds of rating, 27 suggestions were highly supported (median rating 7–9). The most highly rated suggestions concerned diagnostic support (e.g., access to imaging) interventions (e.g., professional or patient education), organisation of the delivery of care (e.g., communication within and between teams) and understanding variations in care and outcomes.

**Conclusions:**

We have identified a set of priorities for implementation research on the early diagnosis of cancer, ranked in importance by primary care physicians and patients. We suggest that researchers and research funders consider these in directing further efforts and resources to improve population outcomes.

**Supplementary Information:**

The online version contains supplementary material available at 10.1186/s12913-023-10330-z.

## Background

Cancer is the leading cause of death in the United Kingdom (UK) [1] with projections indicating approximately 500,000 new cases per year by 2035 [2]. Early diagnosis is associated with better outcomes. For example, 57% of patients with lung cancer survive for 5 years or longer when diagnosed at Stage I, compared to only 3% of those diagnosed at Stage IV [3]. Primary care is of key importance in diagnosis as most (two-thirds) patients will initially present with symptoms in this setting [4]. Early diagnosis is also more cost-effective for healthcare systems [5].

Substantial efforts have gone into promoting earlier diagnosis, including in the UK, with the development of national guidance [6] and establishment of urgent referral pathways. Retrospective studies suggest that these initiatives have resulted in improved detection rates. Neal et al. [7] found a reduction in the period between first presented symptom in primary care and date of subsequent diagnosis (the diagnostic interval) following the introduction of national guidelines, with considerable variations according to the types of cancer. Round et al. [8] found that greater use of the urgent referral pathway was associated with more cancers detected. Moreover, higher practice referral rates are associated with reduced cancer mortality [9], potentially through cancers being diagnosed at an earlier stage [8].

However, the translation of emerging research findings into routine clinical practice can be a slow and haphazard process. There are well-recognised variations in health care and outcomes, including in the timely diagnosis of cancer [10, 11]. Much variation can be considered as “unwarranted” insofar as it cannot be solely attributed to differences in patient characteristics. There are recognised gaps in the implementation of clinical guidelines, in referral rates and the use of investigations although the extent and impacts of unwarranted variations are not fully known. A recent comparison of data from the National Cancer Diagnosis Audit in England in 2018 and 2014 [12] showed that many elements of the primary care cancer diagnostic process had improved substantially in the period since the introduction of NICE guidance on the referral of suspected cancer in primary care [6]. However, the use of diagnostic tests (e.g., faecal immunochemical tests) in primary care had changed very little over this period, despite the NICE recommendations. Others have observed substantial between-practice variation in primary care use of gastrointestinal endoscopy [13].

Implementation research is the scientific study of methods to promote the translation of research evidence into routine clinical practice, and thereby improve the quality of care [14]. It involves designing and evaluating approaches to change the behaviour of healthcare organisations and professionals. Research priorities have been identified for the early detection of cancer but not specifically for implementation research [15]. Setting an agenda for such research in cancer diagnosis needs to consider how to maximise value and minimise research waste [16] (while ensuring patient safety). It is important to include end-users of research in priority setting in order to ensure relevance and increase the likelihood of impact. We therefore undertook a multi-stage consensus development process to identify priorities for implementation research on cancer diagnosis in primary care.

## Methods

Consensus development methods are widely used in producing clinical guidance, designing interventions, and agreeing research priorities [17–19]. Examples of the latter include critical care [20] and oncology [21–23]. They generally make use of participants’ relevant experience and knowledge of a topic area to rate a series of items. We based our approach on the remote RAND/UCLA modified Delphi panel method [17]. We chose this approach over other consensus development methods as it allows participants to complete their ratings independently before meeting to discuss thought processes and areas of disagreement [19].

### Generation and grouping of suggestions for implementation research

We conducted an online survey (open March to May 2021) to elicit suggested priorities for implementation research to improve the diagnosis of cancer in primary care (see Supplementary File 1). We emailed invitations to participate in the survey, including a web-link to the survey, via the distribution lists of the UK Society for Academic Primary Care, the Cancer and Primary Care Research International Network, the CanTest Collaborative (an international network of primary care cancer researchers investigating early diagnosis [24]), members of the Policy Research Network in Cancer Awareness, Screening, and Early Diagnosis, and our existing public and patient involvement panel which had previous experience of participation in consensus processes [25]. We encouraged recipients to share the survey with interested colleagues.

The survey invited respondents to suggest any topics they believed contained scope for improved implementation (“should be done more often or introduced”) or for de-implementation (“be done less often or stopped”). We provided examples of implementation research topics to help respondents distinguish these from clinical research topics. Our examples included: safety netting for patients with concerning symptoms or negative tests; keeping clinicians up to date with pathways for suspected cancer; and increasing or decreasing certain types of referrals for suspected cancer. We placed no limits on the number of suggestions submitted. We asked respondents for information on their roles and locations and whether they would be amenable to a follow-up telephone call to discuss their suggestions. These discussions contributed to clarification and editing of suggestions.

We collated and grouped suggestions. This included merging or removing duplicate suggestions (e.g., multiple respondents suggested simplifying referral processes) and excluding any that were less relevant to implementation research (e.g., developing more precise diagnostic tools). We combined some items, e.g., suggestions around providing patient education on symptoms of concern, risk factors, and screening attendance were combined into a single item that included these examples as a footnote.

We reviewed our listed suggestions in the light of other identified research priorities in cancer detection [15] to check whether our survey had missed any relevant to implementation research.

### Consensus process

We convened a panel to consider the suggestions for implementation research generated by the survey. We aimed for 11 members as consensus panels gain relatively little in reliability by exceeding this number [19]. We approached potential members via existing networks, including respondents from the initial survey, primary care physicians with known interests in research (although not necessarily in cancer research), and an existing public and patient involvement panel with experience of clinical research and living with cancer. The panel comprised nine primary care physicians (including some with regional cancer leadership roles) and two public and patient representatives. Panel membership was deliberately weighted towards primary care physicians because they would typically be targeted by implementation research in this field.

We emailed panellists a link to a summary of the suggestions for implementation research and questionnaire. Panellists independently rated the degree to which they considered the suggestions were a priority for implementation research on a single nine-point scale (9 = very high priority; 1 = very low priority).

We encouraged participants to rate every suggestion, whilst allowing them to indicate if they felt unable to rate a particular suggestion. We also provided space for optional free text comments below each group of suggestions for panellists to explain the rationale behind their scoring. We also invited panellists to add any suggestions that they felt had been overlooked. Panellists completed their first round of rating between May and August 2022. We collated panellist ratings and calculated the median score and range for each suggestion. We defined moderate disagreement if two panellists rated a suggestion towards both ends of the scale, i.e., two panellists scored 1–3 and two panellists scored 7–9. We defined high disagreement if at least three panellists rated a suggestion towards both ends of the scale.

We convened a panel meeting in November 2022, remotely via Microsoft Teams. Before the meeting we emailed all panellists personalised results of the first round of rating. For each suggestion, we provided their own initial rating and the panel median and range. The results indicated levels of disagreement using a traffic light system (green for no disagreement; amber for moderate disagreement; red for high disagreement). The panel meeting was facilitated by RF, who has experience of chairing similar discussions [25–27]. Each suggestion was considered in turn, with particular focus upon those with moderate to high levels of disagreement. The discussion provided an opportunity to clarify the suggestions and elaborate reasons behind high or low ratings. Immediately after considering each suggestion, we asked panellists to re-rate them; panellists who had omitted scoring suggestions on the first round were allowed to score them if they wished on this second round. TW and RN attended the meeting and provided further clarification of questionnaire items and the clinical context, where necessary. We gave panellists unable to attend the meeting an opportunity for a briefing discussion with TW to clarify any suggestions and cover any key points from the remote meeting.

The second round of ratings were submitted during November 2022; we collated responses and calculated median scores. We defined medians of 7 to 9 as indicating strong support for a research suggestion, 4 to less than 7 as moderate support and 1 to less than 4 as weak support.

## Results

### Generation and grouping of suggestions for implementation research

There were 32 respondents to the online survey (Table 1); 12 were primary care professionals, 11 were researchers, and a further four had combined primary care and research roles. Four respondents were patients with personal or familial experience of cancer diagnosis. Most respondents (88%) were based in England. Follow-up conversations were completed with eighteen respondents. The respondents provided 115 suggestions in total. After merging duplicates, we reduced this to 37 items grouped within 17 categories.

**Table 1:** Demographic detail of survey respondents

Role		Location	
Primary care physician (PCP)	11	England	26
Other primary care staff	1	Scotland	2
Consultant	1	Republic of Ireland	1
Patient/public	4	Denmark	2
Researcher	11	Australia	1
PCP & researcher	4		
**Total**	**32**		**32**

We identified no missed suggestions from the earlier, wider priority-setting exercise but modified the wording of some of our suggestions [15].

### Consensus process

Eight panellists attended the discussion meeting, and one (a primary care physician) took part in an individual briefing meeting. Both panellists that did not attend a meeting were primary care physicians. All 11 panellists completed first and second rounds of rating. After the first round, there was high disagreement around six suggestions and moderate disagreement around two. After the second round, the number of suggestions with high disagreement remained at two whilst the number with moderate disagreement rose to 14.

After the second round of rating, 27 suggestions were strongly supported (i.e., median rating over 7), and within this group eleven suggestions had a median rating of 8 or above. Nine suggestions were moderately supported, and one weakly supported (Tables 2, 3 and 4). The overall spread of support was similar between the first and second rounds of ratings. Within these classifications, however, three suggestions moved from high to moderate support and three from moderate to high support. Levels of support did not change for the remaining 31 suggestions.

**Table 2:** Suggestions with strong support (i.e., final median value of seven or higher) and disagreement level

Category	Suggestion	Median score (R1)	Median score (R2)	Disagreement
Diagnostic tools: imaging	Primary care access to cancer-relevant imaging technologies, e.g., diagnostic ultrasound, or equipment to take high-quality dermatoscopy photos that can be sent electronically to secondary care	9	9	-
Analysis of existing data	Exploring existing data from patients with cancer to look for patterns or early warning signs that might have been missed (e.g., analysis of repeat prescriptions as a potential risk indicator)	8	8	-
Communication within and between teams	Exploring the best way to co-ordinate information between different healthcare sectors and professionals to improve the early detection of cancer	8	8	
Management of vague symptoms	Design and implementation of processes to support the management of patients with vague or imprecise symptoms, and the impact of these processes upon subsequent investigations and referrals	8	8	
Variations in care	Analysis of variations in cancer care or outcomes	8	8	-
Patient education	Patient information and education campaigns	8	8	-
Referral processes, care pathways and organisation of care	Review of cancer-specific referral pathways to identify potential improvements. This might include *within* individual pathways (e.g., identifying potential bottlenecks), as well as moving *between* them if a diagnosis subsequently changes.	8	8	Moderate
Clinician education	Continuing education for primary care staff around cancer-relevant topics. This could include information on treatment and referral pathways, and individual risk factors, for example	9	8	-
Risk stratification	Incorporate a cancer risk check into NHS Health Check. This might include blood tests such as an emerging Multi-Cancer Early Detection (MCED) test	8.5	8	-
Resource use and wider consequences	Exploration of the harms or unintended consequences of a focus on early detection and diagnosis. This might involve evaluation of local or national initiatives	7	8	-
Diagnostic tools: access and recording	Access to point of care diagnostics	6	8	-
Clinical guidelines	GP involvement in guideline design to promote clarity and relevance	7.5	8	Moderate
Variations in care	Exploring consistency in the interpretation of presented information, e.g., research using standard videos	8	7	-
Decision support tools	Supporting decision making regarding possible cancer in patients at the extremes of age	7	7	-
Resource use and wider consequences	Health economic analysis of approaches to early detection and diagnosis of cancer. For example, the cost-effectiveness of increasing access to diagnostic tests, or of changes to clinical pathways	7	7	-
Diagnostic tools: access and recording	Including specific blood tests that may detect cancer early within routine care for other conditions	6	7	-
Care pathways: follow-up care	Guidance and processes for ongoing follow-up of patients after a cancer diagnosis	7	7	-
Safety netting	Exploring whether and how safety netting procedures might be improved	7	7	-
Decision support tools	Understanding barriers to clinician use of computerised cancer risk tools	7	7	Moderate
Risk stratification	Use of genetic testing to identify individuals at risk of developing cancer	7.5	7	-
Risk stratification	Use of machine learning to support targeted screening and reduce overscreening	7	7	-
Patient involvement	Approaches to patient communication	8	7	Moderate
Organisation of care	Follow-up calls, texts or video messages to patients who have not responded to screening invitation	7	7	Moderate
Decision support tools	Primary care access to decision support tools (e.g., QCancer) during consultations. Such tools may be designed to pull data from within the patient record, update in response to new information or test results, and help to identify patients in need of referral or further testing	7	7	Moderate
Referral processes, care pathways and organisation of care	Use of Faecal Immunochemical Test to prioritise bowel cancer referrals	5	7	Moderate
Risk stratification	Primary care access to risk assessment tools for risk stratification and risk-stratified cancer early detection and prevention. This might permit the delivery of targeted advice on risks	7	7	-
Management of vague symptoms	Physical examination of patients with persistent abdominal / gastro-intestinal symptoms	7	7	Moderate

Moderate disagreement defined as two or more panellists rating the suggestion 1–3 and two or more rating it 7–9

**Table 3:** Suggestions with moderate support (i.e., final median values of four to six) and disagreement level

Category	Suggestion	Median score (R1)	Median score (R2)	Disagreement
Referral processes, care pathways and organisation of care	Exploring alignment of local Two Week Wait suspected cancer pathways to NICE guidance	6	6	-
Safety netting	Understanding of the barriers to patients coming back following a consultation	7	6	-
Decision support tools	Exploration of how computerised decision support tools align with local criteria	7	6	Moderate
Diagnostic tools: access and recording	Templates to specify the reasons for any blood tests completed as well as their results	6	6	Moderate
Referral processes, care pathways and organisation of care	Access to and use of patient referral systems	6	6	High
Decision support tools	Evaluation of the use of cancer mind maps	7	5	Moderate
Diagnostic tools: imaging	Use of Artificial Intelligence to examine primary care imaging outputs	5	5	Moderate
Organisation of care	Local cancer ‘champions’ with responsibility for reminding primary care teams about referral pathways, awareness of new guidance, etc.	6	5	Moderate
Organisation of care	Organising general practices to ensure that one key clinician is kept up to date with pathways for suspected cancer	6	5	High

Moderate disagreement defined as two or more panellists rating the suggestion 1–3 and two or more rating it 7–9; high disagreement defined as three or more panellists rating the suggestion 1–3 and three or more rating it 7–9

**Table 4:** Suggestions with weak support (i.e., final median value below four) and disagreement level

Category	Suggestion	Median score (R1)	Median score (R2)	Disagreement
Organisation of care	Initial meetings with nurses / nurse-led clinics to explore patient symptoms, before passing to a GP who can then work with that information	3	3	Moderate

Moderate disagreement defined as two or more panellists rating the suggestion 1–3 and two or more rating it 7–9

The 27 suggestions showing strong support covered a range of topics. Several suggestions concerned diagnostic support (e.g., access to imaging technologies such as ultrasound or dermatoscopy, and other point of care diagnostic tools; access to risk stratification or decision support tools; guidance on the management of vague symptoms). Educational interventions, for patients (e.g., information campaigns) and clinicians (e.g., continuing professional development on topics such as referral pathways or individual risk factors), also received strong support, as did suggestions relating to the structure and organisation of delivery of care (e.g., communicating information within and between teams; review of referral pathways to identify potential improvements) and analysis of variations in care and outcomes.

## Discussion

Our structured and transparent approach has identified and ranked a set of priorities for implementation research to improve the early diagnosis of cancer in primary care. The suggestions with strongest support included proposals concerning access to imaging and diagnostic tools, educational interventions, improving information communication between teams, and understanding variations in care and outcomes. We have built upon existing research priorities for cancer diagnosis [15] by focusing on research to understand and change professional and organisational behaviour and hence close gaps between clinical evidence and routine care. Our participant sample was weighted in favour of primary care physicians given that they would typically be targeted by much implementation research aiming to change clinical behaviour around this topic. Identifying the priorities of clinicians and patients is important as their priorities may diverge from those of researchers [28].

Some of our priorities are consistent with those identified in earlier priority setting [15, 29] and were relevant to implementation research (e.g., the potential use of computerised-decision support tools to help clinicians recognise patients who repeatedly present with symptoms that may be associated with cancer) as well as more clinically-focused priorities (e.g., the development of new screening tests). Harris et al. [29] analysed the suggestions of 1300 primary care practitioners from 20 European countries on how speed of diagnosis in cancer in primary care might be improved. Greater access to relevant imaging technologies (e.g., diagnostic ultrasound) and improved communication between healthcare teams (e.g., between primary and secondary care) featured prominently and were also highly rated by our panel. Neither of these studies included patients in priority setting.

We highlight four main study limitations. First, our suggested research priorities were mainly generated by self-selected survey respondents. Nevertheless, our sample comprised relevant, experienced stakeholders with backgrounds in primary and secondary care, implementation research, and patient representation, as well as respondents with experience of healthcare outside the UK. Moreover, we took existing priorities into account in generating our suggestions for the consensus panel [15] and offered panellists the opportunity to add any omissions. Second, our suggested priorities were mainly generated and then considered by people with experience of primary healthcare and research in the UK. Our priorities may need to be adapted for or applied cautiously to other healthcare systems, for example, those with less prominent primary care ‘gatekeeper’ functions. Third, we noted that the number of suggested priorities with moderate disagreement increased from six to 14 following the panel meeting. This is counterintuitive as consensus development processes would typically be expected to reduce disagreement; this has been shown to apply to virtual meetings as well as in-person [17]. The virtual meetings reported by Broder et al. were considerably longer (6–7 h), albeit with substantially more items under consideration, and it is possible that our two-hour, online meeting may have limited the time and quality of interactions that would otherwise have allowed panellists to reach agreement. However, our relatively efficient methods did not ‘force’ consensus and there were low levels of disagreement for most research priorities with strong support. Fourth, there is likely to be more of a continuum rather than a dichotomy between ‘clinical research’ and ‘implementation research’. This is perhaps reflected in priorities such as improving access to imaging or using artificial intelligence to help interpret imaging, where the clinical evidence base may need to be strengthened before investigating how to increase adoption and spread in routine healthcare.

We share our ranked list of priorities in the hope that it will be useful to implementation researchers with interests in improving cancer diagnosis in primary care and research funders. It reflects the perspectives of patients and health professionals who are typically the end-users of implementation research.

However, suggested research priorities concerning early diagnosis of cancer must be considered in the context of a changing primary care landscape. The COVID-19 pandemic had a substantial impact upon cancer detection, with significant reductions in the use of cancer diagnostic procedures, referrals, and diagnoses (e.g., [30, 31]). The pandemic compounded the existing pressures on the primary care system. Any newly developed interventions should ideally seek to contribute to reducing the existing patient backlog and be compatible with primary care processes and ways of working revised in response to the pandemic. They should also align with the recently announced changes to cancer waiting times standards [32]. Black et al. [33] have recently outlined how a ‘systems approach’ may help to improve the early diagnosis of cancer within primary care. This perspective shifts the emphasis away from interventions that target individual elements of the diagnostic process or consultation (e.g., clinician education or decision support tools) and instead embraces the complexity of the care system, acknowledging the many different steps where potential delays or errors may occur. For instance, automated processes to share information (e.g., test results) between relevant team members, and use of technology to detect missed or incomplete actions and thus reduce unnecessary delays. Our initial survey identified suggestions for implementation research that were consistent with this systems-level approach, and some of these were strongly supported by our panel, e.g., improving communication within and between teams, and the review of referral pathways to identify areas for improvement.

There is heightened awareness of disparities within care quality and outcomes and this is recognised by the recent NHS England strategy, Core20PLUS5 [34]. The strategy’s aim is to improve care for the most deprived 20% of the population and address locally identified inequalities through quality improvement. Our survey item, “analysis of variations in cancer care or outcomes” may implicitly suggest a need to reduce inequalities and was strongly supported by the panel. Demographic and socioeconomic factors have been regularly associated with differential care and outcomes, for example in terms of screening uptake [35], likelihood of diagnostic delay [36] and both experience of care and prognosis [37]. It is important that any interventions arising from this prioritisation exercise include their impact upon inequalities in subsequent evaluation.

It is also worth considering those suggestions that received substantially lower levels of support (while noting that these tended to have moderate or high levels of disagreement between panel members). The lowest supported items were from the category “organisation of care” and concerned either ensuring key individuals (‘champions’) within each practice were up to date with relevant cancer information or introducing initial meetings with nurses to explore symptoms before passing this information to a primary care physician. Panel discussions suggested that the former approach had been tried before and was often unsustainable, by focusing upon single individuals as opposed to more widespread change, whilst the latter represented inefficient use of scarce resources for minimal benefit.

## Conclusion

This consensus process has identified a set of priorities for implementation research with the aim of improving the early diagnosis of cancer in primary care. Primary care professionals and patients provided insights into what they considered important for research in this field, including better access to imaging and diagnostic technologies, educational interventions for professionals and patients, organisation of the delivery of care and communication between teams, and understanding variations in care and outcomes.

### Electronic supplementary material

Below is the link to the electronic supplementary material.

Supplementary Material 1: Online survey text

## Data Availability

The datasets analysed during this study are available from the corresponding author on reasonable request.

## List of abbreviations

NHS: National Health Service
PCP: Primary care physician
UK: United Kingdom
