# Supplementary material for: Priorities for implementation research on diagnosing cancer in primary care: a consensus process
Source: BMC Health Serv Res. 2023 Nov 27;23:1308. doi: 10.1186/s12913-023-10330-z (PMC10683096; doi:10.1186/s12913-023-10330-z)
Supplement: Supplementary file 1 — Supplementary Material 1: Online survey text [file 12913_2023_10330_MOESM1_ESM.docx]

**Identifying implementation priorities for early detection and diagnosis of cancer in primary care**

Thank you for your interest in this study.

We are researchers at the University of Leeds, working with the Department of Health and Social Care Policy Research Unit for cancer awareness, screening and early diagnosis. We are seeking to improve the detection and early diagnosis of cancer in primary care.

We are currently generating a list of potential priorities for research to improve the early detection and diagnosis of cancer in primary care. We have invited a range of clinicians, patients and other stakeholders to draw upon as wide a range of experience and knowledge as possible.

On the next page you will be provided with some further guidance about the task and space to enter your suggestions. Your participation in this study is entirely voluntary and you are free to withdraw at any time. However, please note that if you do withdraw from the study it may not be possible to remove data that has been submitted.

The study has been reviewed and given a favourable opinion by the School of Medicine Research Ethics Committee at the University of Leeds (ref: MREC 20-054).

## **Suggested priorities**

We are interested in anything that you believe should be done **more often or introduced**, as well as anything that you feel should be done **less often or stopped**.

Please try to consider any issue relating to patients in primary care. **Examples** of types of things that you could suggest include:

- Safety-netting for patients with concerning symptoms or negative tests
- Keeping clinicians up to date with pathways for suspected cancer
- Increasing or decreasing certain types of referrals for suspected cancer

There is no limit to the number of suggestions that you can make.

You do not have to provide a reason for your choices, although any explanatory detail would be useful.

Please enter your suggestions in the space below.

|  |
| --- |

## **A little detail about you**

We would be grateful if you could tell us a little about yourself. The following two questions ask for basic information about your location and your role. Both questions are optional but this detail would be helpful to us.

Where do you live? Please select from the following options.

England

Scotland

Wales

Northern Ireland

Other (please state)

How would you describe your role? Please choose from the options available.

General Practitioner / Family Doctor

Other primary care staff

Consultant

Other secondary care staff

Patient / public

Researcher

Other (please state)

## **Contact details**

It is possible that we would like to discuss your suggestions further to clarify details and ensure understanding.

If you would be happy to complete a short telephone call (30 minutes maximum), please provide your contact details below and we will arrange a convenient time to speak.

Name

Email address

Telephone number *Optional*
